# Evaluation of gut microbiota predictive potential associated with phenotypic characteristics to identify multifactorial diseases

**DOI:** 10.1080/19490976.2023.2297815

**Published:** 2024-01-18

**Authors:** Danielle Cristina Fonseca, Ilanna Marques Gomes da Rocha, Bianca Depieri Balmant, Leticia Callado, Ana Paula Aguiar Prudêncio, Juliana Tepedino Martins Alves, Raquel Susana Torrinhas, Gabriel da Rocha Fernandes, Dan Linetzky Waitzberg

**Affiliations:** a Laboratory of Nutrition and Metabolic Surgery of the Digestive System, LIM 35, Department of Gastroenterology, Hospital das Clínicas HCFMUSP, Faculdade de Medicina, Universidade de São Paulo, São Paulo, Brazil; b Department of Gastroenterology, Hospital das Clínicas HCFMUSP, Faculdade de Medicina, Universidade de São Paulo, São Paulo, Brazil; c Biosystems Informatics and Genomics Group, Instituto René Rachou - Fiocruz Minas, Belo Horizonte, Brazil

**Keywords:** Gut microbiota, phenotypic variables, 16S rRNA, random forest, prediction models

## Abstract

Gut microbiota has been implicated in various clinical conditions, yet the substantial heterogeneity in gut microbiota research results necessitates a more sophisticated approach than merely identifying statistically different microbial taxa between healthy and unhealthy individuals. Our study seeks to not only select microbial taxa but also explore their synergy with phenotypic host variables to develop novel predictive models for specific clinical conditions. Design: We assessed 50 healthy and 152 unhealthy individuals for phenotypic variables (PV) and gut microbiota (GM) composition by 16S rRNA gene sequencing. The entire modeling process was conducted in the R environment using the Random Forest algorithm. Model performance was assessed through ROC curve construction. Results: We evaluated 52 bacterial taxa and pre-selected PV (*p* < 0.05) for their contribution to the final models. Across all diseases, the models achieved their best performance when GM and PV data were integrated. Notably, the integrated predictive models demonstrated exceptional performance for rheumatoid arthritis (AUC = 88.03%), type 2 diabetes (AUC = 96.96%), systemic lupus erythematosus (AUC = 98.4%), and type 1 diabetes (AUC = 86.19%). Conclusion: Our findings underscore that the selection of bacterial taxa based solely on differences in relative abundance between groups is insufficient to serve as clinical markers. Machine learning techniques are essential for mitigating the considerable variability observed within gut microbiota. In our study, the use of microbial taxa alone exhibited limited predictive power for health outcomes, while the integration of phenotypic variables into predictive models substantially enhanced their predictive capabilities.

## Significance of this study

**What is Already Known on this Subject?**

- While the gut microbiota has been implicated as potential signatures or biomarkers for various clinical conditions, the establishment of causality in humans remains largely elusive.
- The role of the gut microbiota in maintaining the host organism’s proper physiological function is well-established, yet data regarding the composition of the gut microbiota in disease states often suffer from poor reproducibility.

**What Are the New Findings?**

- Our study demonstrates that relying solely on differences in the relative abundance of bacterial taxa between groups falls short as a means of identifying clinical markers.
- We advocate the use of robust statistical tools, such as bootstrapping, to mitigate the substantial variability observed in gut microbiota studies, thereby enhancing the reproducibility of research findings.
- Our findings underscore the limited predictive power of microbial taxa in isolation for health outcomes.
- The integration of phenotypic variables into predictive models with gut microbiota significantly augments the ability to predict health outcomes.

**How This Study Might Advance Research**

- Despite the growing enthusiasm for using gut microbiota as biomarkers for various clinical conditions, the lack of standardization throughout the research process impedes progress in this field.
- Our study emphasizes the necessity of rigorously testing predictions of clinical conditions based on gut microbiota using bootstrapping techniques, promoting greater reproducibility in research findings.

## Introduction

The human microbiome represents a vast and intricate consortium of microorganisms that colonize the human body. Comprising archaea, bacteria, fungi, and viruses, these diverse inhabitants reside in various anatomical niches. Of particular focus in contemporary research, the gut microbiota (GM) has garnered significant attention for its extensive study.^1^

The qualitative and quantitative composition of the gut microbiota (GM) has frequently been proposed as a promising indicator of both health and disease states.^2^ However, the intricate web of intra- and inter-individual diversities within the human gut microbiota complicates the direct utilization of intestinal bacteria as exclusive markers for the prediction of health and disease states.^3^

Numerous studies have identified bacteria with varying relative abundances between control and diseased groups as potential markers for disease states such as inflammatory bowel disease,^4,5^ systemic lupus erythematosus,^6^ type 1^7^ and type 2 diabetes.^8^ However, these findings often lack consensus and reproducibility among different studies, making the practical application of these bacteria as reliable markers challenging.^9,10^ Some authors have sought to create indices based exclusively on bacterial taxa for predicting health outcomes,^2^ while others emphasize the importance of associating host information to enhance predictive capacity.^11^

Recent studies employing robust machine learning techniques have also shed light on this issue. They suggest that the gut microbiota, in isolation, exhibits limited discriminatory power in distinguishing between individuals in good health and those facing health challenges.^12^

In the past two decades, extensive research has delved into the multifaceted physiological functions of the gut microbiota. However, despite this growing body of work, our comprehensive understanding of how the gut microbiota contributes to predicting multifactorial diseases remains a persistent challenge. It is important to emphasize that an effective biomarker should translate biological observations into clinically relevant outcomes. Good biomarkers are characterized by low variability and reliable responsiveness to changes in the assessed condition..^13^ While the identification of microbial taxa with differing relative abundances is a starting point, it may not necessarily represent a biomarker^14^. These taxa can be considered as candidates and require rigorous evaluation through robust predictive models that account for specificity and sensitivity.^9^

The primary aim of this study is to develop predictive models that integrate phenotypic variables with gut microbiota taxa to accurately distinguish between individuals in good health and those facing health challenges. Subsequently, we will rigorously evaluate the performance of these integrated predictive models.

## Methods

### Study design and participants

We conducted a single-center cross-sectional case-control study, enrolling a total of 202 participants. This study was a subset of a larger research initiative, VALIDYS (CAAE: 01713018.0.0000.0068 - CAPPesq 3,008,966), which recruited participants between December 2018 and February 2020. Prior to their participation, all individuals provided written informed consent in accordance with the Declaration of Helsinki guidelines.

#### Inclusion and exclusion criteria

The recruitment of study participants occurred for convenience and followed specific inclusion and exclusion criteria, ensuring that the selected individuals met the predefined parameters.

#### Inclusion criteria of control group (CG)

- Self-reported healthy and asymptomatic adults.
- No continuous use of medication (except oral contraceptives for women).
- Age: 18 to 80 years.

- BMI (Body Mass Index): 18.5 to 24.99 kg/m^2^.
- No complaints of any infection or disease.

#### Inclusion criteria for disease group

- Age: 18 to 80 years.
- Confirmed diagnosis of one of the following conditions, being in follow-up care at the respective units of the Hospital das Clinicas of the University of São Paulo (HCFMUSP): Type 2 Diabetes (T2D), Type 1 Diabetes (T1D), Plaque Psoriasis (PP), Systemic Lupus Erythematosus (SLE) in clinical remission, Rheumatoid Arthritis (RA), Inflammatory Bowel Diseases (IBD) – Crohn’s Disease (CD) or Ulcerative Colitis (UC) in clinical remission, being in follow-up care at the Division of Gastroenterology and Hepatology and the Division of Coloproctology of HCFMUSP.

#### Exclusion criteria for both groups

- Pregnant individuals.
- Subjects with pacemakers and stents.
- Subjects currently undergoing therapeutic treatment with antineoplastics and antibiotics.

- Use of antibiotics within the last 3 months.
- Presence of chronic diseases, including HIV, Chronic Kidney Disease (CKD), Cirrhosis, Acromegaly, and hyperthyroidism.
- A history of cancer and anticancer treatment within the last 10 years.

#### Data anonymization

To safeguard the privacy and personal data of the participants and adhere to data protection laws, all participant data were declassified and anonymized.

## Data collection

### Phenotypic and clinical data collection

Clinical anamnesis was conducted by our research team to obtain a comprehensive dataset from the patients. The information collected encompassed the following aspects:

- Demographic Information: Date of birth, ethnicity, sex, marital status, education, profession/occupation.
- Medical History: History of current and previous illnesses.
- Lifestyle Factors: Smoking, alcohol consumption.
- Allergies and Food Intolerance: Presence of allergies or food intolerances.
- Physical Activity: Information on physical activity levels.
- Dietary Habits: Usual food consumption, including fruits, vegetables, greens, processed foods, and consumption of sugars/sweeteners.
- Bowel Habits: Assessment of bowel habits, including the frequency of bowel movements and adherence to the Bristol stool scale.
- Nutritional Supplements and Medications: Details on the use of nutritional supplements and medications, including type, frequency, and dosage.

### Nutrient habitual consumption assessment:

Food intake data were meticulously collected using a 24-hour dietary recall (24 HR), administered on three separate occasions before and after stool sample collections. The food items reported in each of the 24 HR were initially recorded in cooking units, such as tablespoons. Subsequently, our research team standardized and converted these units to grams or milliliters for consistency.

The energy intake, macronutrients, and total dietary fiber were quantified using the Easydiet software, which incorporates the Brazilian Table of Food Composition (TACO)(Brazilian food composition table (TACO) n.d.)and the Table of Food Composition: Support to Nutritional Decision (*Table for evaluating food consumption in culinary measurements* 2004). To estimate the usual energy and nutrient intakes, the Multiple Source Method (MSM) was employed. This method was applied through the online platform available at https://msm.dife.de/.

#### Fecal sample collection

To ensure uniformity in the collection process, all participants were provided with a standardized kit for fecal sample collection. Detailed instructions were given for the proper procedure:

1. Participants used a sterile swab to transfer a small fecal sample into 2 ml plastic microtubes pre-filled with a DNA preservation buffer solution.
2. Participants were advised to collect the stool sample no more than 24 hours before delivering it to the research team.
3. The collected fecal samples were to be stored at a controlled temperature (refrigeration) until the moment of delivery to the research team.

#### Fecal sample processing and DNA extraction

DNA extraction from fecal samples was conducted utilizing the QIAamp® PowerFecal® DNA Kit by QUIAGEN® in accordance with a standardized protocol (*Illumina. 16S Metagenomic sequencing library preparation*. n.d.).

#### Extraction procedure

- Averaging at 0.25 grams of sample, the fecal samples were homogenized in 2 ml tubes containing metallic microspheres. This procedure facilitated cell lysis of both host and microbial cells. Complementing this, the addition of appropriate chemical reagents ensured the efficient extraction of bacterial DNA.
- The DNA extraction kit employed Inhibitor Removal Technology (TRI) specifically tailored for stool samples. This technology effectively eliminated inhibitory substances commonly found in fecal material, such as polysaccharides, heme, and bile salts, which can interfere with subsequent polymerase chain reaction (PCR) processes.
- Following successful DNA extraction, the total genomic DNA was captured on a silica membrane within a column format. Subsequent washing and elution processes were carried out, resulting in the isolation of DNA with high purity, ready for genetic sequencing of the microbiota.

#### Library preparation and 16S rRNA sequencing

The library preparation involved the application of unique identifiers to each DNA sample. These identifiers allowed for the simultaneous processing of all samples, streamlining the sequencing process. In this step, specific primer sequences were added, following the Illumina® protocol, which included variable regions V3 and V4. The primer sequences used were as follows:

#### 16S amplicon PCR forward primer

5’TCGTCGGCAGCGTCAGATGTGTATAAGAGACAGCCTACGGGNGGCWGCAG 3’

#### 16S amplicon PCR reverse primer

5’GTCTCGTGGGCTCGGAGATGTGTATAAGAGACAGGACTACHVGGGTATCTAATCC 3’

The protocol also incorporated overhang adapter sequences, which were appended to the primer pair sequences to ensure compatibility with the Illumina index and sequencing adapters.

#### Sequencing preparation

Subsequently, the prepared samples were pooled, and the libraries were quantified through PCR, employing Quant-iT and Qubit DNA quantitation assay kits (PICOGREEN®, Quant-iT™), as well as Kapa Hifi Hotstart (Roche®). These quantification steps ensured precise sample measurement before introducing them into the sequencer.

#### Gut microbiota data processing

The raw sequence reads of the 16S rRNA gene underwent thorough processing, trimming, and assembly, leading to the generation of amplicon sequence variants (ASVs). This process was accomplished using DADA2.^15^ During the processing, the primers used in amplification were systematically removed, and sequences with more than two expected errors were eliminated, ensuring data integrity. The remaining sequences were employed to create an error identification and correction model, enhancing data accuracy. The forward and reverse readings, already corrected, were concatenated to form the ASVs. This concatenated data was used to eliminate chimeric sequences and to quantify the ASVs accurately.

Each ASV received a taxonomic classification via the TAG.ME package.^16^ This classification was based on the SILVA database as a reference, utilizing the specific model tailored for the amplicon corresponding to the 341F-805 R region.

#### Gut microbiota analysis

The Beta diversity was evaluated by calculating the Bray-Curtis dissimilarity. The results were visualized in a Principal Coordinate Analysis (PCoA). Additionally, PCoA was constructed using Jensen-Shannon divergence and Weighted UniFrac distance. These techniques provided insights into the dissimilarity between microbiota samples. A Permanova test was employed to identify distinctive microbial patterns associated with each disease under study.

To identify differentially abundant bacterial taxa within each clinical group (each unhealthy group compared to the healthy group), the Wald test from the DESeq2 package (Version 1.30.1)^17^ was utilized. During this analysis, candidates were selected based on adjusted p-values for multiple tests using the Benjamini-Hochberg method (p-adjusted *p* < 0.05).

#### Statistical analysis

The Continuous variables were presented as mean and standard deviation or median and interquartile range, while categorical variables were expressed as absolute and relative frequencies. The Shapiro-Wilk test was used to assess the normality of continuous variables.

For continuous variables that did not follow a normal distribution, a non-parametric Mann-Whitney U test was employed when comparing two groups.

Analyses involving three or more groups with non-normally distributed variables were performed using the Kruskal-Wallis test. Subsequently, in cases where the Kruskal-Wallis test indicated significance (*p* < 0.05), the Dunnett posthoc test was applied to compare each specific disease group with the healthy group.

The categorical variables were evaluated using the chi-square test.

A significance level of 0.05 was adopted for all tests, with two-tailed hypotheses considered.

The statistical analyses were conducted using JASP Team software (Version 0.14.1) and R version 4.0.5.

### Creation of predictive models to discriminate between healthy and unhealthy individuals using isolated phenotypic or gut microbiota characteristics

Predictive models were constructed a priori for each set of data, including quantitative, qualitative, and abundance of genera of the microbiota. The entire modeling process was conducted in the R environment using the “caret” package^18^ with the Random Forest algorithm. The selection of the Random Forest algorithm was made following a comparative evaluation of four distinct classification algorithms, which included Generalized Linear Models (GLM), Boosted Logistic Regression (BLR), and Logistic Model Trees (LMT). To assess their overall performance, the Area Under the Curve (AUC) was used as a key metric, with predictions based on microbial abundance to determine disease state. The results of this assessment are presented in **Supplementary Table S1**. The Random Forest algorithm utilized in this study consisted of 100 trees with cross-validation repeated 20 times.

The evaluation of the predictive performance of the models for each disease involved four stages: 1) Utilizing bacterial taxa data exclusively to determine the isolated ability of microbial information to detect one of the diseases under investigation; 2) Assessing the capacity of qualitative phenotypic characteristics to detect one of the diseases; 3) Examining the quantitative phenotypic characteristics’ ability to detect one of the assessed diseases; and 4) Integrating steps 1, 2, and 3 to detect one of the diseases evaluated in this study.

The dataset was randomly divided, with half of the healthy and unhealthy patients used for model building, and the remaining patients employed to test the model and evaluate its performance.

Model performance was evaluated by measuring predictive ability, specifically Sensitivity and Specificity, using the test dataset. Additional performance metrics such as Recall, and Precision were also calculated.

The importance of each variable was assessed using the “Mean Decrease Gini” index^19^, representing the percentage of predictions that the model fails to hit when removing that variable. Additionally, to ensure the robustness and reproducibility of these measurements, SHAP (SHapley Additive exPlanations) values were calculated for each variable. These SHAP values provide valuable insights into the influence of each variable on the model’s predictions. Furthermore, the Pearson correlation between the SHAP values and the “Mean Decrease Gini” index was calculated. This correlation analysis served as an additional validation step, confirming the consistency of variable importance measurements across different approaches.

The entire procedure described above was repeated 50 times, each time involving a random selection of 50 different datasets for training and test data. During each iteration, data on the importance of the variables were recorded, and the top 10 most important variables were identified. The performance of each model was used to construct the Receiver Operating Characteristic (ROC) curve.

### Creation of an integrative predictive model

The integrative predictive model aimed to effectively discriminate between healthy and unhealthy individuals by considering both phenotypic characteristics and gut microbiota. To construct this model, variables that appeared among the top 10 most important in at least half − 25 times – of the models for at least one of the diseases were selected. These variables were consistently present in each dataset, encompassing quantitative, qualitative, and microbiota data. The final integrative model was generated through a consensus approach, combining information from 50 bootstrap resamplings. This approach ensured robustness and reliability. The importance of each variable was meticulously stored, and the model’s performance was assessed by constructing a Receiver Operating Characteristic (ROC) curve. The Area Under the Curve (AUC) was calculated as a measure of the model’s separability capacity. A higher AUC value indicates better discrimination between patients with the disease and healthy controls. The use of Random Forest (RF) modeling provided AUC-ROC values that spanned from random discrimination (AUC = 0.5 or 50%) to perfect discrimination (AUC = 1.0 or 100%).

**Figure 1.**
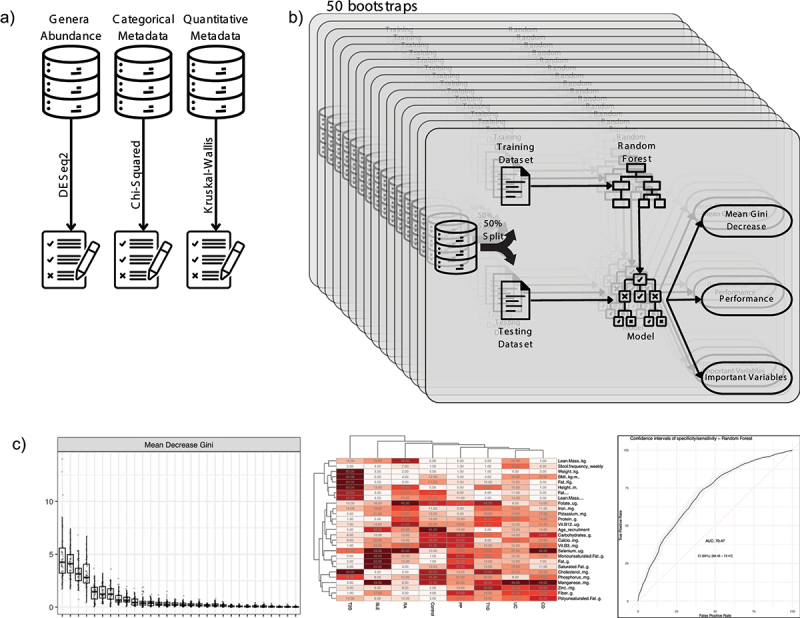
Workflow overview and variable selection process: A) initial datasets and the process of selecting candidate variables using statistical tests: DESeq2, chi-squared and Kruskal-Wallis. B) the bootstrap process repeat 50 times the creation of a random forest model used to variable selection and performance evaluation. C) visualization of three key performance metrics: mean Gini Decrease used to identify the most important variables, variable importance frequency heatmap, and a receiver operating characteristic (ROC) curve representing final model performance.

Figure 1 provides an overview of the workflow, starting from the initial data selection and progressing through multiple bootstraps. The final stage involves the selection of variables for the integrative model.

## Results

### Characteristics of sample

The study population consisted of a total of 202 individuals, comprising 50 healthy controls and 152 individuals with various diseases. Each specific disease group represented approximately 15% of the total diseased sample, with the exception of the Inflammatory Bowel Diseases (IBD) group, which included two subtypes, contributing to 30% of the diseased sample. In terms of age, the control group (CT) had a median age of 32 years, which exhibited significant differences when compared to other disease groups, including Plaque Psoriasis (PP) with a median age of 55 years, Rheumatoid Arthritis (RA) with a median age of 59.5 years, and Type 2 Diabetes (T2D) with a median age of 52.5 years. Gender distribution revealed a higher prevalence of females in all disease categories, while the control group displayed a balanced distribution of individuals of both sexes. A more detailed breakdown of phenotypic characteristics can be found in Table 1.

**Table 1.** Phenotypic characteristics of the study participants.

Variables	CT	IBD	PP	RA	SLE	T1D	T2D	Pvalue
	(*n*=50)	(*n*=40) a	(*n*=20) b	(*n*=20) c	(*n*=20) d	(*n*=20) e	(*n*=32) f	
Age (min-max)	32	40	55	59.5	35.5	35	52.5	<.001 * b.c.f
	(19–83)	(20–72)	(34–71)	(32–69)	(30–45)	(19–56)	(33–73)	
Ethnicity (n)(%)								
Black	2.0 (4.0)	3.0 (7.5)	5.0 (25.0)	3.0 (15.0)	2 (10.0)	1 (5.0)	3 (9.4)	<.001 #
Brown	4.0 (8.0)	14.0 (35)	6.0 (30.0)	4.0 (20.0)	7 (35.0)	8 (40.0)	19 (59.4)	
White	39.0 (78.0)	23.0(57.5)	9.0 (45.0)	13.0 (65.0)	11(55.0)	11 (55.0)	10 (31.2)	
Asian	5.0 (10.0)	0.0 (0.0)	0.0 (0.0)	0.0 (0.0)	0 (0.0)	0 (0.0)	0 (0.0)	
Gender (%)								
Female	23.0 (46.0)	24 (60.0)	12.0 (60.0)	15 (75.0)	20 (100)	11 (55.0)	17 (53.0)	0.003 #
Male	27.0 (54.0)	16 (40.0)	8 (40.0)	5 (25.0)	0 (0.0)	9 (45.0)	15 (47.0)	
BMI (kg/m^2^) (%)								
Malnutrition	0.0 (0.0)	1 (2.5)	0 (0.0)	0 (0.0)	0 (0.0)	1 (5.0)	0 (0.0)	<.001#
Eutrophy	28.0 (56.0)	18 (45.0)	7 (35.0)	9 (45.0)	9 (45.0)	10 (50.0)	7 (21.8)	a.b.c.f
Overweight	21.0 (42.0)	14 (35.0)	7 (35.0)	4 (20.0)	10(50.0)	7 (35.0)	3 (9.4)	
Obesity	0.0 (0.0)	7 (17.5)	6 (30.0)	6 (30.0)	1 (5.0)	2 (10.0)	22 (68.8)	
Body composition								
Weight (kg)	68	70	72	68	65.5	71	97	<.001*f
	(41.5–100)	(43–108)	(49.1–106)	(47.5–92)	(51.5–82)	(40–97)	(58–201)	
BMI (kg/m^2^)	24.3	25.4	27	25.6	25.4	24.9	37.5	<.001*f
	(18.1–29.8)	(17.8–5.9)	(20.2–7.5)	(20.8–6.6)	(19.4–31.6)	(16.7–31.3)	(21.6–60.6)	
Fat mass (%)	26.8	33	39.2	44.2	33.2	29.1	47.5	<.001*
	(8.7–41.0)	(17–48.6)	(20.3–5.4)	(31.7–1.7)	(19.6–46.8)	(15.6–42.9)	(19.7–68.1)	a.b.c.f
Lean mass (%)	73.2	66.95	60.8	55.8	66.9	70.9	52.5	<.001* a.b.c.f
	(59.0–91.3)	(51.4–3.0)	(44–79.7)	(38.3–8.3)	(50.7–80.4)	(57.1–84.4)	(31.9–80.3)	

Phenotypic characteristics and nutritional habits are detailed in **Supplementary Tables S2 and S3**, respectively. Food intake was categorized into healthy food consumption, less healthy food consumption, and alcohol consumption. All statistically significant differences were preselected for model construction.

Additionally, the habitual nutrient intake values were calculated based on the results obtained using the MSM residues method, which involved three 24-hour dietary recall (24 HR) for each individual within each evaluated group. The averages for each group can be found in **Supplementary Table S4**. Notably, when comparing with the control group, certain nutrients, including carbohydrates, polyunsaturated fat, fiber, sodium, zinc, vitamin A, and vitamin D, showed no significant differences in their habitual intake.

Values are presented as median (minimum-maximum). Differences between both groups were analyzed using the Mann-Whitney U test* or chi-squared test^#^. Values are significant when *P* < 0.05. Abbreviations: CT: control group; IBD: inflammatory bowel disease; PP plaque psoriasis; RA rheumatoid arthritis; SLE: Systemic lupus erythematosus; T1D: type 1 diabetes; T2D: type 2 diabetes; BMI: Body mass index.

### Microbial community structure and clinical status

**Figure 2.**
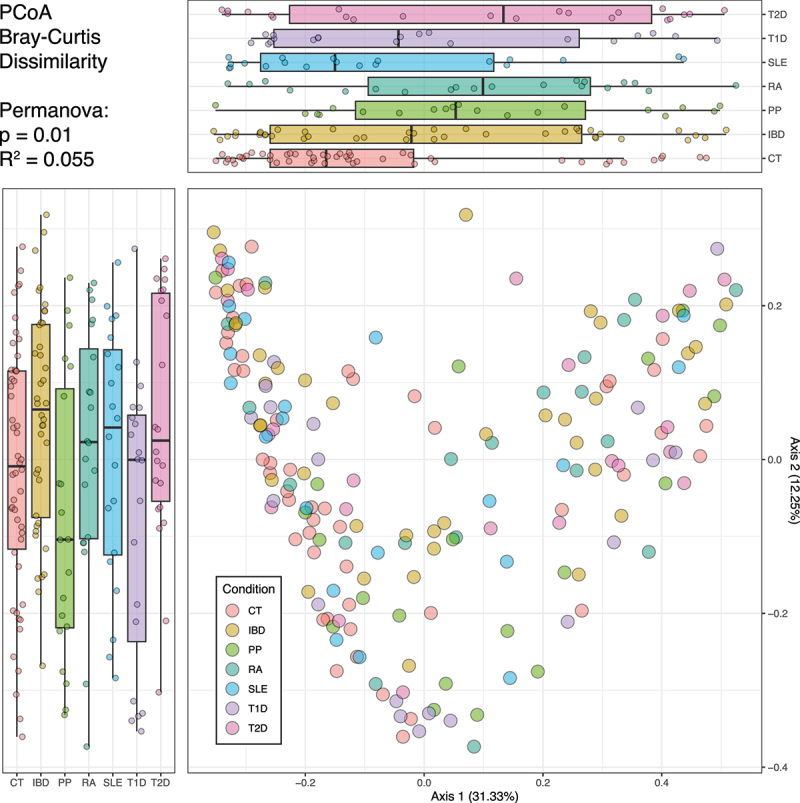
Beta diversity representation of gut microbiota in health and clinical conditions. the scatter plot displays the distribution of individuals, with axis 1 on the X-axis and axis 2 on the Y-axis. Each point on the scatter plot is color-coded to indicate the individual’s health or disease status. Boxplots are presented alongside the scatter plot for each health or disease condition. These boxplots depict the spread and central tendency of individuals across both axis 1 and axis 2.

The analysis of the gut microbiota’s macrostructure among different groups involved the comparison of β diversity metrics (Figure 2). Using principal coordinates analysis (PCoA) based on Bray-Curtis dissimilarity, we found that Axis 1 represented 31.33% of the variance, while Axis 2 accounted for 12.55% of the variance. Notably, the figure highlights the substantial overlap of points among all health and disease conditions in both axes, suggesting that the gut microbiota’s beta diversity does not distinctly separate individuals based on their health or disease status. This visualization underscores the complex and multifactorial nature of the microbial community structure in relation to clinical conditions. Additionally, despite the Permanova test yielding a p-value of 0.01, the R^2^ value indicated that disease status could only explain approximately 5.5% of the inter-individual variation. The same overlapping pattern could be identified in the PCoA using two other different distance metrics – the Jensen-Shannon divergence and the Weighted UniFrac distance – and are shown in **Supplementary Figure S1**.

### Selection of candidate variables

The pre-selected variables, which included microbial taxa identified as differentially abundant between disease and healthy subjects, as well as phenotypic variables identified through statistical tests, were used as candidates for model construction. These pre-selected variables are presented in Supplementary Figure S2.

Subsequent to the pre-selection of the aforementioned phenotypic and microbial variables, a Random Forest filtering process was employed to finalize the variable selection. This procedure involved 50 repetitions, where 50 datasets were randomly generated for training and test data. The variables that consistently emerged among the 10 most important variables, as assessed by Mean Gini Decrease, were further examined for their frequency across distinct subpopulations.

To enhance the reproducibility of the results and gain deeper insights into variable importance, we also employed the SHAP (SHapley Additive exPlanations) analysis. Mean SHAP values were calculated for each variable and subsequently correlated with the Mean Gini Decrease values across the 50 bootstrap rounds. The high correlation index (Rho) observed when correlating the same variables indicated that the variable maintained its order of importance, regardless of the measurement method used. Conversely, comparing different variables yielded Rho values approaching zero, signifying distinct importance rankings. The values of Mean Gini Decrease, Mean Absolute SHAP values, and the distribution of correlation values can be found in **Supplementary Figure S3**. This comprehensive approach further strengthened the reproducibility of our results while providing valuable insights into variable significance.

**Figure 3.**
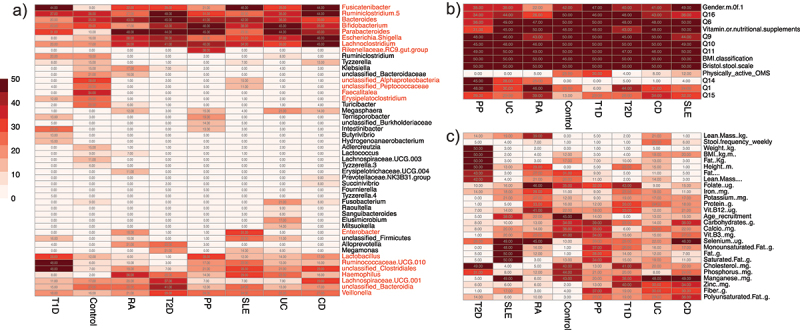
Heatmaps of variables frequency among important markers. A) frequency of the bacterial taxa among the selected variables. Taxa denoted in red met the inclusion criteria for the final model by appearing in at least 25 bootstraps for at least one health or disease state. B) frequency of qualitative phenotypic variables. All variables within this category were chosen for the final model. C) frequency of quantitative phenotypic variable. All variables in this category were selected as they met the selection criteria for at least one disease state.

Several genera, including Fusicatenibacter, Ruminoclostridium 5, Bacteroides, Bifidobacterium, Parabacteroides, Escherichia-Shigella, and Lachnoclostridium, were frequently observed among the selected variables, indicating their potential as markers for various disease states. Within the quantitative variables, we observed a sporadic distribution of frequencies, with notable signatures linked to variables such as adipose composition and BMI-related metrics (weight and height) associated with Type 2 Diabetes. In contrast, most of the qualitative variables exhibited higher frequencies, primarily due to the limited number of variables tested. The findings are visually presented in Figure 3, which features a heatmap illustrating the frequency of each tested variable among the 10 most important variables in each of the 50 bootstraps. Variables that recurred in at least half of the simulations, a total of 25 times, were selected for the construction of the final predictive model.

### Ability of isolated gut microbiota to differentiate disease states and the control group

The selected taxa that met the criteria of being relevant for prediction in at least 25 subpopulations were utilized to construct a refined model. The predictive potential of the gut microbiota varied among different diseases, with some autoimmune-related conditions showing weaker performance, including Rheumatoid Arthritis (RA) with an AUC of 54.19, Ulcerative Colitis (UC) with an AUC of 52.34, and Systemic Lupus Erythematosus (SLE) with an AUC of 49.08. In contrast, Crohn’s Disease and Plaque Psoriasis exhibited slightly better predictive performance, achieving AUC values of 69.54 and 65.95, respectively. Notably, the gut microbial composition demonstrated greater accuracy in predicting diabetes, with an AUC of 72.65 for Type 2 Diabetes and 78.91 for Type 1 Diabetes.

Intriguingly, the sensitivity for Ulcerative Colitis was unexpectedly low at 27%, although this was offset by a high specificity of 82%. A similar trade-off between recall and precision was observed in the case of Rheumatoid Arthritis, with a sensitivity of 86% and precision of 11%. Detailed performance metrics for all disease states are provided in **Supplementary Table S5**.

### Enhanced model accuracy through phenotypic variable integration

The inclusion of phenotypic variables in the predictive model significantly enhanced the model’s accuracy for all diseases. Notably, we observed a substantial improvement in the Systemic Lupus Erythematosus (SLE) model, which initially achieved an AUC of 49.08 when relying solely on microbial variables. However, with the integration of phenotypic variables, the SLE model’s performance remarkably increased to 98.4. This improvement was particularly prominent in the case of nutritional variables, which played a pivotal role in the model, accounting for the six most decisive variables.

The significance of anthropometric variables was particularly evident in the Type 2 Diabetes model, where relying solely on microbial information yielded an AUC of 72.65. However, the incorporation of anthropometric variables substantially increased the AUC to 96.96, underlining their crucial role in enhancing model performance.

While the integration of phenotypic variables proved pivotal, the microbial community’s importance remained evident. In the case of the Plaque Psoriasis model, the initial AUC of 65.95 improved to 76.02 with the integration of additional variables, yet it retained three microbial taxa as the most influential variables.

The Table 2 provides a comprehensive comparison of the isolated AUC and integrated AUC for each disease and details on the ten most important variables in each integrated model.

**Table 2.** Comparison of performance of disease predictive models using isolated and integrated gut microbiota and phenotypic variables.

Disease	Isolated AUC disease	Integrated AUC	Ten most important markers in the integrated models
	(level of discrimination)	(level of discrimination)	
CD	69.54 (regular)	75.25 (good)	1 Manganese (ni)
			2 Selenium (ni)
			3 Carbohydrates (ni)
			4 Fusicatenibacter (bt)
			5 Rumminoclostridium 5 (bt)
			6 Lachnoclostridium (bt)
			7 Vitamin B3 (ni)
			8 Polyunsaturated fat. (ni)
			9 Fiber (ni)
			10 Escherichia-Shigella
UC	52.34 (weak)	60.42 (regular)	1 Manganese (ni)
			2 Bifidobacterium (bt)
			3 Zinc (ni)
			4 Fibers (ni)
			5 Escherichia-Shigella (bt)
			6 Parabacteroides(bt)
			7 Lachnoclostridium (bt)
			8 Fusicatenibacter (bt)
			9 Ruminiclostridium 5 (bt)
			10 Bacteroides (bt)
PP	65.95 (regular)	76.02 (good)	1 Parabacteroides (bt)
			2 Bifidobacterium (bt)
			3 Rikenellaceae RC9 (bt)
			4 Calcium (ni)
			5 Carbohydrates (ni)
			6 Fiber (ni)
			7 Monounsaturated fat. (ni)
			8 Saturated fat (ni)
			9 Vitamin B3 (ni)
			10 Selenium (ni)
RA	54.19 (weak)	88.03 (excelent)	1 Selenium (ni)
			2 Vitamin B12 (ni)
			3 Folate (ni)
			4 Protein (ni)
			5 Lean Mass (cc)
			6 Iron (ni)
			7 Potassium (ni)
			8 Height (cc)
			9 Fat mass (cc)
			10 Bacteroides (bt)
SLE	49.08 (weak)	98.4 (excelent)	1 Selenium (ni)
			2 Total fat. (ni)
			3 Monounsaturated fat. (ni)
			4 Saturated fat (ni)
			5 Manganese (ni)
			6 Vitamin supplement (ni)
			7 Age (cc)
			8 Bacteroides (bt)
			9 Fusicatenibacter (bt)
			10 Bifidobacterium (bt)
T1D	78.91 (good)	86.19 (excelent)	1 Folate (ni)
			2 Cholesterol (ni)
			3 Ruminococcaceae UCG 010 (bt)
			4 unclassified_Clostridiales (bt)
			5 Zinc (ni)
			6 Fusicatenibacter (bt)
			7 Protein (ni)
			8 Monounsaturated fat (ni)
			9 Manganese (ni)
			10 Vitamin B12 (ni)
T2D	72.65 (good)	96.96 (excelent)	1 BMI (cc)
			2 Weight (cc)
			3 Fat mass (cc)
			4 Height (cc)
			5 Cholesterol (ni)
			6 Lean Mass (cc)
			7 Fat mass (cc)
			8 Phosphorus (ni)
			9 Selenium (ni)
			10 Lachnospiraceae UCG 001 (bt)

*AUC discrimination levels according to Hosmer and Lemeshow.*^20^ *AUC: area under the curve; CD: Crohn’s disease; UC: Ulcerative colitis; PP, plaque psoriasis; RA, rheumatoid arthritis; SLE, systemic lupus erythematosus; T1D, type 1 diabetes; T2D, type 2 diabetes; (ni): nutrient intake; (bt).: bacterial taxon; (cc): body composition or clinical feature. The table describes the 10 most important variables for predictive models*

In summation, our findings underscore the potential of integrating both phenotypic information and microbial characteristics as a comprehensive approach to enhance the prediction of health conditions. This holistic perspective offers valuable insights for advancing predictive modeling in the context of multifactorial diseases.

## Discussion

In our study, we set out to explore the potential of gut microbiota, both in isolation and in conjunction with phenotypic information, for identifying individuals with various clinical conditions through machine learning. This approach represents a shift away from the conventional evaluation of “dysbiosis,” which typically focuses on differences in relative bacterial abundance between control and specific disease groups.^21–23^

Our study encompassed a total of 202 individuals, with approximately 20 participants in each subgroup for every specific disease under investigation. It is worth noting that the sample size in studies involving the gut microbiota exhibits substantial variability. This variability often arises due to factors such as accessibility to clinical populations, budget constraints, and the nature of the research question. In this context, our study stands out as it operates within the context of a larger sample, allowing for an in-depth examination of multiple diseases, each with a sizable representation. These variations in the sample size afford our investigation a broader spectrum of subjects, enhancing the generalizability of our findings.

One unique aspect of our study is the considerable variability in the phenotypic profiles of our participant cohort. While many studies often employ strict filtering criteria based on factors such as age, gender, ethnicity, BMI, and dietary habits to reduce potential impacts on the gut microbiota, our work embraced this diversity. We view this diversity as an advantage, as it enabled us to select phenotypic variables that ultimately contributed to our predictive models. These differences within our dataset allowed us to identify what is most pertinent for predicting health outcomes, aligning with the perspective outlined in a review on “experimental and computational considerations to support the reproducibility of studies involving microbiomes.”^10^

Our study unveiled an intriguing phenomenon in the context of gut microbiota macrostructure, specifically in terms of beta diversity. Figure 2 illustrates a considerable overlap among individuals in different disease groups, challenging the notion of clear-cut distinctions between these conditions. However, a Permanova analysis revealed that, despite the lack of significant explanatory relevance, the only factor displaying a significant association with the gut microbiota macrostructure was the health or disease status.

Advancements in genome sequencing and bioinformatics have undoubtedly expanded our understanding of the intricate host-microbiome relationships. Nevertheless, since the initial publications highlighting differences in the intestinal microbiome across various clinical conditions^6,24–26^, the field has grappled with considerable heterogeneity in findings regarding bacterial composition in these diseases. The absence of a distinct “healthy” gut microbiota profile further complicates the overarching comprehension of these intricate relationships.^27^

Understanding the microbial profile alone provides limited insight into the intricate landscape of autoimmune and metabolic diseases, which predominantly manifest as multifactorial conditions. In this context, it becomes increasingly imperative to complement microbial data with information about phenotypic characteristics, particularly those associated with diet, lifestyle, and body composition.^28^ Wilkinson and colleagues emphasized the substantial potential in studying the human microbiota on a population scale in a recent review. They underscored the significant implications for public health, particularly in the realm of identifying novel biomarkers, therapeutic approaches, or molecular mechanisms. This exploration begins with observational studies involving human subjects and proceeds to more detailed characterization in experimental settings.^29^

Machine learning, often denoted as Machine Learning (ML), plays a pivotal role in microbiome data analysis, contributing to enhanced work reproducibility, the development of predictive models with potential diagnostic applications, and serving as a foundation for clinical interventions related to microorganisms or their modulation.^30–33^

Predictive models have emerged as valuable tools in the realm of gut microbiota research, primarily aimed at evaluating their efficacy in diagnosing conditions and predicting responses to treatments. Decision tree algorithms are commonly employed for such purposes, given their capacity to deduce the outcome variable from the provided data used for model training. These decision trees serve as a fundamental framework for implementing other machine learning techniques, including the random forest.

Random forest (RF) stands out as a prominent bootstrap aggregation algorithm that has found utility in various studies involving the gut microbiota. One example is in the classification of pediatric patients with Crohn’s disease (CD), wherein RF algorithms were employed to discern disease status and treatment response based on alpha diversity and genetic risk scores.^34^ Surprisingly, 16S rRNA datasets outperformed shotgun metagenomics, demonstrating the robust classification capabilities of RF in microbiome research.^35,36^

An insightful study conducted by Ananthakrishnan and colleagues^37^ offers valuable parallels to our own investigation. They explored the potential of gut microbiome characteristics in predicting the response to biologic therapy with vedolizumab among patients afflicted with Crohn’s disease and ulcerative colitis. The methodology adopted in their research shares similarities with our approach. Initially, they developed a model using clinical data in isolation, which yielded modest performance in predicting expected outcomes, with an AUC of 62% (representing a regular capacity).

In contrast, when microbial taxa were integrated into their model, a notable improvement was observed, resulting in an AUC of 72%, indicative of good predictive capability. However, the most impressive performance was achieved when the model was holistically integrated, combining clinical data with the gut microbiota’s taxonomic information. This synergistic approach yielded the best performance with an AUC of 78%, demonstrating a good predictive capacity. This compelling outcome underscores the power of combining clinical and microbiome data to enhance predictive models and their clinical applications.

Despite the differing primary objectives of the study by Ananthakrishnan and colleagues,^37^ it is noteworthy that our research shares some intriguing parallels. Notably, in both studies, the integration of clinical and gut microbiota data outperformed the utilization of these datasets in isolation. In our cohort, utilizing microbial information in isolation resulted in a regular predictive performance, with an AUC of 69.54%. However, when we integrated phenotypic and microbiota data into a single model, we achieved a markedly improved performance, with an AUC of 75.25%, mirroring the performance attained by Ananthakrishnan and his team.

On the other hand, in the case of predictive models for ulcerative colitis (UC) within our cohort, we observed performance levels close to random chance, characterized by limited performance both in isolation and through an integrated approach (AUC = 52.34% and AUC = 60.42%, respectively). This outcome suggests that, for the specific profile of individuals in our cohort previously described by Rocha et al.,^38^ the gut microbiota may not be the most reliable marker for distinguishing healthy individuals from those with UC or the other diseases under consideration. These findings emphasize the intricate and context-dependent nature of the host-microbiome interplay in different clinical conditions, underscoring the necessity of considering the specific disease context in microbiome-related research.

Wu et al.^39^ conducted a comprehensive study involving Chinese subjects to assess the predictive potential of gut microbiota in distinguishing between four distinct conditions: rheumatoid arthritis, type 2 diabetes, liver cirrhosis, and a healthy control group. Their analysis, which incorporated metagenomic data of the microbiota, yielded promising results with an AUC of 81% for type 2 diabetes, an impressive AUC of 94% for rheumatoid arthritis, and a strong AUC of 83% for liver cirrhosis.

In the context of lupus (systemic lupus erythematosus, or SLE), recent investigations have also explored the utility of microbial information in predicting disease status. Wei et al.^40^ delved into this area and constructed a predictive model utilizing bacterial genera as potential biomarkers for SLE. Their model, comprising nine genera, exhibited robust discriminatory power with an AUC of 74% for distinguishing SLE patients from healthy controls. In contrast, our research, focusing on gut microbiota as an isolated variable, produced performance levels akin to random chance, with an AUC of 49.08%. Furthermore, the bacterial taxa that emerged as important variables in our model differed from those identified in the studies conducted by Wei et al. and others.

These divergent outcomes underscore the disease-specific nature of the relationship between gut microbiota and clinical conditions. Variations in population, genetic factors, and environmental influences may contribute to these differences, emphasizing the need for tailored approaches to microbiome research in distinct disease contexts.^28^

Li et al.^41^ conducted a study to distinguish between systemic lupus erythematosus (SLE) patients and both healthy controls and rheumatoid arthritis (RA) patients based on the genus and species composition of gut bacteria. Their predictive model, considering bacterial taxa such as Lactobacillus mucosae, Megasphaera, and Streptococcus enriched in SLE, along with reduced Faecalibacterium in healthy controls, achieved a performance with an AUC of 79%, a result quite comparable to Wei’s findings.^40^

It’s plausible that the more modest differences observed in our study compared to Li^41^ and Wei^40^ could be attributed to the specific characteristics of our study population. Our cohort of SLE patients was previously described by Balmant et. al^42^ and consisted of clinical remission individuals, all of whom had a SLEDAI score of 0, and comprised entirely of female participants. It’s noteworthy that SLE generally exhibits a milder disease severity in women compared to men, which may explain the discrepancies in the microbial profiles and their associations with disease status.

These insights highlight the importance of accounting for the heterogeneity of disease characteristics within study populations, as this variability can significantly impact the relationship between gut microbiota and clinical conditions, ultimately affecting predictive modeling outcomes.

The literature regarding the association between gut microbiota (GM) and Type 1 Diabetes (T1D) in adults is relatively limited, with only a handful of studies addressing this topic. One of the most recent investigations by Shilo et al.^7^ delved into the predictive role of GM in T1D in adults. In their study, they found that adults with T1D exhibited a distinct microbial profile that allowed for the development of a high-capacity predictive model, with an impressive AUC of 0.89. They identified elevated levels of specific bacteria, including *Prevotella copri* and *Eubacterium siraeum*, alongside reduced *Faecalibacterium prausnitzii* in these individuals.

In our own study, when we considered microbial variables in isolation, the predictive model for T1D yielded a good performance (AUC = 78.91%). However, we observed a substantial improvement in predictive accuracy (AUC = 86.19%) when we incorporated information about nutrient consumption, particularly regarding folate, cholesterol, zinc, manganese, and protein. These nutrients are known to exert a significant impact on glycemic control and can also influence the systemic inflammatory profile of the individual.^43^ The interplay between these factors underscores their importance in the management of T1D and offers a plausible explanation for the enhanced performance of our predictive model when this nutritional information was integrated into the dataset.

These findings emphasize the complex interrelationships among GM, dietary factors, and metabolic conditions, particularly in the context of T1D. They also highlight the potential utility of an integrated approach, where both microbial and nutritional data are considered in tandem to enhance the accuracy of predictive models for T1D.

Type 2 Diabetes (T2D) remains a focal point in the field of gut microbiota research, and numerous studies^24,44,45^ have strived to uncover a distinct microbial profile in individuals affected by the condition. However, these efforts have thus far failed to yield a unified microbial signature associated with T2D. In our current study, when evaluating the performance of a predictive model for T2D using solely gut microbiota data, we observed a performance with an AUC of 72.65%.

In contrast, our integrated model, which combined phenotypic characteristics with microbial markers, exhibited exceptional performance, achieving an impressive AUC of 96.96%. This substantial improvement in predictive accuracy underscores the significance of considering both phenotypic data and the gut microbiota when aiming to identify T2D. It also reinforces the idea that T2D is a multifaceted condition influenced by a wide array of factors, both microbial and phenotypic, which collectively contribute to its complex etiology.

The challenges encountered in pinpointing a consistent microbial profile for T2D across various studies highlight the intricate nature of this disease and its potential heterogeneity among affected individuals. The success of our integrated model highlights the importance of adopting a comprehensive approach that integrates diverse data sources, offering a promising path toward improving T2D prediction and enhancing our understanding of the condition’s multifactorial underpinnings.

The recent study by Gou et al.^46^ adds to the growing body of research on Type 2 Diabetes (T2D) and its association with the gut microbiota. In their investigation, Gou and colleagues assessed the predictive accuracy of various factors, including microbiome characteristics, host genetic information, and additional phenotypic features such as cardiovascular risk scores, lifestyle factors, and dietary habits. Their findings revealed that utilizing microbiome data outperformed the other factors in predicting T2D.

Specifically, Gou et al.^46^ observed that a predictive model based solely on phenotypic characteristics achieved an AUC of 63%. However, when microbiota data were integrated into the model, the AUC significantly improved to 73%. This outcome aligns with our own study, reinforcing the notion that incorporating microbiome information alongside lifestyle characteristics can substantially enhance the predictive capability of machine learning models.

These findings collectively emphasize the significance of adopting a holistic approach that integrates microbiome data and various aspects of an individual’s lifestyle to bolster the accuracy of T2D prediction models. The ability of the gut microbiota to contribute substantially to predictive accuracy underscores its relevance in understanding and managing the multifaceted nature of T2D and other complex metabolic conditions.

The identification of microbial biomarkers for predicting various diseases and conditions has become a significant area of interest within the scientific community. In several instances, such as the enrichment of *Fusobacterium nucleatum* in patients with colorectal cancer (CRC),^47–49^ existing research findings have demonstrated remarkable convergence. This consensus exists irrespective of geographic regions, lifestyle variations, age disparities, and other potentially confounding factors. These instances of agreement across studies reinforce the reliability of certain microbial biomarkers as valuable indicators of specific health conditions.

However, it is essential to acknowledge that for other health conditions, the path to identifying robust microbial biomarkers is more complex. In these cases, despite notable advancements through the integration of omics-based techniques and computational methodologies, the reproducibility of potential microbial biomarkers often remains a challenge. The variability in findings across different studies can lead to concerns regarding the reliability and generalizability of these biomarkers for diagnostic and predictive purposes.

As the field of microbiome research continues to evolve, researchers must address these complexities and work toward enhancing the consistency and reproducibility of microbial biomarker discovery. Additionally, as highlighted in our study and corroborated by others, integrating microbial data with phenotypic characteristics, lifestyle factors, and dietary habits can significantly enhance the predictive performance of machine learning models. This holistic approach may represent a promising direction for improving the accuracy and reliability of microbial biomarkers in diverse health conditions.

The mounting evidence supporting the role of the microbiome in human health is substantial. However, the rush for expedited findings within the field of integrative microbiomics, often lacking standardized protocols for sample collection, handling, extraction, sequencing, and analytical methodologies, as well as the incorporation of essential clinical data, presents significant obstacles. These challenges impede the establishment of robust scientific conclusions and contribute to the propagation of less reproducible results.^10,29,33^

As a constructive recommendation to mitigate bias in future studies within this field, our research underscores the significance of employing variable filtering, both phenotypic and microbial, through resampling techniques such as bootstrapping. This approach can help mitigate the impact of substantial variability that’s often observed in gut microbiota studies and promote the reproducibility of results obtained.^10^

Microbial taxa, therefore, should be considered as candidates for predicting specific conditions and rigorously tested for their reproducibility and discriminatory capacity through predictive models, as exemplified in this study.

## Conclusions

While certain microbial taxa (genera and species) do exhibit varying relative abundance between groups, they do not, in isolation, possess predictive marker potential. The evaluation of beta diversity fails to reveal a distinct pattern within the microbiota macrostructure that can effectively discriminate between health status and disease states.

Collectively, our findings emphasize that relying solely on microbial markers for predicting health outcomes is limited in its effectiveness. However, integrating phenotypic characteristics with gut microbiota data yields an enhanced predictive model that improves the predictability of health outcomes.

## Supplementary Material

Supp_Fig_4.eps

Supp_Table_4.xlsx

Supp_Table_5.xlsx

Supp_Fig_2.eps

Supp_Table_2.xlsx

Supp_Table_3.xlsx

Supp_Fig_1.eps

Supp_Fig_3.eps

Supp_Table_1.xlsx

## Data Availability

Nucleotide sequence data used for this study are deposited in The European Nucleotide Archive (ENA) accession number PRJEB59338.
